# Methylation data from *Pseudotaxus chienii* obtained using methylation-dependent restriction-site associated DNA sequencing

**DOI:** 10.1016/j.dib.2018.06.045

**Published:** 2018-06-26

**Authors:** Yingjuan Su, Zhen Wang, Ting Wang

**Affiliations:** a School of Life Sciences, Sun Yat-sen University, Guangzhou 510275, China; b Research Institute of Sun Yat-sen University in Shenzhen, Shenzhen 518057, China; c College of Life Sciences, Nanjing Agricultural University, Nanjing 210095, China; d College of Life Sciences, South China Agricultural University, Guangzhou 510642, China

**Keywords:** Methylation, MethylRAD, *Pseudotaxus chienii*

## Abstract

*Pseudotaxus chienii* is an endangered coniferous plant that is endemic to China. Because *P. chienii* is sessile and has a long life cycle, its options for responding to drastic or rapid changes in climate are limited. To survive locally, *P. chienii* must be able to adapt, and the species shows variations in leaf size along an environmental gradient from east to west. It is important to determine whether this phenotypic variation is driven by DNA methylation. Therefore, we performed a preliminarily survey using methylation-dependent restriction-site associated DNA sequencing (MethylRAD) to investigate the methylation status of three *P. chienii* individuals from heterogeneous ecological niches. In total, 372,611 CCGG tags and 726,332 CCHGG tags were obtained. The rate of high quality methylation tags for a specific site in the genome varied from 42.31% (Gxdms3-4) to 50.01% (Jxbj3-4) and 50.18% (Zjdxg3-6). The level of CCHGG methylation (16.63%) was higher than that of CCGG (13.60%), which may be why *P. chienii* has low levels of phenotypic variation. The methylation data can be accessed using the Sequence Read Archive (SRA) database (SRP128155).

**Specifications Table Methylation**

**Table:** 

Subject area	Biology
More specific subject area	*Pseudotaxus chienii,* Methylation
Type of data	Tables
How data was acquired	MethylRAD
Data format	Clean
Experimental factors	Three individuals from heterogeneous ecological niches
Experimental features	Genomic DNA was extracted from *Pseudotaxus chienii* and digested with FspEI. Sample barcodes were introduced. Single-end sequencing was performed using an Illumina Hiseq X Ten sequencer.
Data source location	Gxdms3-4 (23°29′54′′ N; 108°26′12′′ E)
	Jxbjs3-4 (26°30′35′′ N; 114°09′41′′ E)
	Zjdxg3-6 (27°52′49′′ N; 119°10′24′′ E)
	All the three individuals were kept at School of Life Sciences, Sun
	Yat-sen University.
Data accessibility	The Methylation data have been deposited and made accessible via BioProject ID: PRJNA419098; BioSample accessions: SAMN08048873, SAMN08048874, and SAMN08048875; and the SRA database (SRP128155).

**Value of the data**

• This dataset provides valuable information that could help predict epigenetic adaptations to future changes in climate.
• These data enhance our understanding of the mechanisms of natural phenotypic variation and may be used to provide guidance for the management of genetic resources and species conservation.

## 1 Data

This article provides methylation data for three *Pseudotaxus chienii* individuals from heterogeneous ecological niches. The clean data were deposited in the National Center for Biotechnology Information SRA database (SRP128155).

## 2 Experimental design, materials and methods

### 2.1 DNA samples and digestion

In this study, we selected one *P. chienii* individual from Daxiagu in Zhejiang Province (Zjdxg3-6), one from Bijiashan in Jiangxi Province (Jxbj3-4), and one from Damingshan in Guangxi Zhuang Autonomous Region (Gxdms3-4). *P. chienii* individuals across these regions possess high genetic diversity and show strong local adaptations to rapid environmental changes, which are suitable for methylation analysis [1]. Young and healthy leaves at the same developmental stage and from the same climate conditions were sampled. We extracted genomic DNA using a modified cetyltrimethylammonium bromide method [2]. Each genomic sample (200 ng) was digested separately with FspEI (New England BioLabs, cat. no. R0662L) at 37 °C for 45 min together with control DNA [3].

### 2.2 Adaptor ligation, amplification, and purification

A 20 µL ligation mix that included ligation master mix, 5 µM adaptor A, 5 µM adaptor B, and digested DNA was incubated at 16 °C for 1 h. The sequences of the two adaptors were as follows:

slx-ada1-BsaXI: 5′–CAAGCAGAAGACGGCATACGACCGCGCGAGTNNN–3′.

3′–GGCGCGCTCA–5′.

slx-ada2-BsaXI: 5′–CGACAGGTTCAGAGTTCTACAGTCCGACGATCNNN–3′.

3′–TGTCAGGCTGCTAG–5′.

Ligation products were amplified in 30.4 µL of reaction mix containing 8 µM of each primer (p1: 5′–ACACTCTTTCCCTACACGACGCT–3′; and p2: 5′–GTGACTGGAGTTCAGACGTGTGCT–3′), 1.6× High-Fidelity DNA Polymerase Buffer, 0.39 mM dNTPs, 0.4 U of Phusion High-Fidelity DNA polymerase (New England Biolabs, cat. no. M0530, Ipswich, MA, USA), and 18 µL of ligated DNA. PCR amplification was performed for 1–16 cycles using the following conditions: 98 °C for 5 s, 60 °C for 20 s, 72 °C for 10 s, and a final extension step of 5 min at 72 °C. The PCR products were separated using 8% polyacrylamide gel electrophoresis along with a 100-bp DNA ladder (New England BioLabs, cat. no. N3231). The target bands were excised, the DNA was allowed to diffuse out of the gel in nuclease-free water for 30 min at 37 °C and then amplified using the PCR conditions described above. PCR products derived from the three individuals were mixed, further purified, eluted, and quantified.

### 2.3 Barcoding and library pooling

Sample barcodes were introduced using PCR. Each 85-µL PCR reaction mix contained 5× High-Fidelity DNA Polymerase Buffer, 10 mM dNTPs, 10 µM Primer3, 10 µM Index Primer, 1.6 U Phusion High-Fidelity DNA polymerase (New England Biolabs, cat. no. M0530, Ipswich, MA, USA), and 50 ng of gel-extracted PCR product. A total of 1–16 cycles of PCR were performed as described above, and following purification, the PCR products were subjected to single-end sequencing (100–150 bp) using an Illumina Hiseq X Ten sequencer (Illumina Inc., San Diego, California, USA) (Table 1, Table 2).

**Table 1:** Quantity of sequencing data and ratios.

**Samples**	**Clean data**	**MethylRAD tags**	**Quantity of data**	**Ratio**
Gxdms3-4	161,720,241	25,378,549	793,164,733	10,738,505 (42.31%)
Jxbj3-4	161,720,241	24,823,170	776,428,065	12,414,623 (50.01%)
Zjdxg3-6	161,720,241	23,267,447	727,701,890	11,676,360 (50.18%)
Average	161,720,241	24,489,722	765,764,896	11,609,829 (47.50%)

**Table 2:** Summary of methylation site coverage.

**Sample**	**CCGG**		**CCWGG**	
	**Number of sites**	**Average depth**	**Number of sites**	**Average depth**
Gxdms3-4	242,013	13.34	462,085	15.53
Jxbj3-4	256,668	13.31	521,409	16.69
Zjdxg3-6	224,294	14.16	461,613	17.66
Average	240,992	13.60	481,702	16.63
